# Daily Challenge/Hindrance Demands and Cognitive Wellbeing: A Multilevel Moderated Mediation Model

**DOI:** 10.3389/fpsyg.2021.616002

**Published:** 2021-03-08

**Authors:** Huangen Chen, Hongyan Wang, Mengsha Yuan, Shan Xu

**Affiliations:** ^1^School of Business Administration, Southwestern University of Finance and Economics, Chengdu, China; ^2^School of Economics and Management, Chengdu Sport University, Chengdu, China

**Keywords:** challenge demands, hindrance demands, work–family enrichment, perceived overqualification, cognitive wellbeing, diary study

## Abstract

Based on the challenge-hindrance stressor model, this study explored the mechanism of how challenge/hindrance demands affect cognitive wellbeing on a daily basis. Specifically, we examined the mediating effect of work–family enrichment on the relationship between challenge/hindrance demands and cognitive wellbeing. In addition, we tested the moderating effect of overqualification on the relationship between challenge/hindrance demands and work–family enrichment on a daily basis. Finally, we examined the moderated mediation effect of perceived overqualification in a multilevel model. To capture changes in work–family enrichment and cognitive wellbeing that individuals perceived daily, the experience sampling method was adopted to test our theoretical models. A total of 99 participants from China were involved in this investigation. The results showed that daily challenge demands had a significant positive effect on daily cognitive wellbeing, and daily hindrance demands had a significant negative effect on wellbeing. In addition, daily work–family enrichment mediated the positive relationship between daily challenge demands and daily cognitive wellbeing. Moreover, perceived overqualification moderated the relationship between daily challenge demands and daily cognitive wellbeing in the multilevel model. Finally, a significant moderated mediating effect of this overqualification on the indirect effect of daily work–family enrichment on the relationship between daily challenge demands and daily cognitive wellbeing was observed.

## Introduction

Subjective wellbeing refers to an individual’s subjective perception of happiness and represents the overall evaluation of an individual on his/her work and family life (Luhmann et al., 2012b). This wellbeing encompasses affective wellbeing and cognitive component wellbeing. Cognitive wellbeing is an important dimension of employee wellbeing as it is used to describe an individual’s global life satisfaction, which permeates his/her work and family life (Dierendonck, 2004; Horn et al., 2004). Existing studies indicate that employees’ job demands have a negative correlation with their cognitive wellbeing (Anja et al., 2010; Demerouti and Bakker, 2011; Lamb and Kwok, 2016). However, other studies argue the opposite, noting that increased job demands can help improve an employee’s cognitive wellbeing (Anja et al., 2010; Klassen and Chiu, 2010). Although existing research is able to confirm that job demands play a significant role in affecting cognitive wellbeing, consistent conclusions have yet to be reached. The inconsistency here is a reminder of the necessity of distinguishing between different types of job demands, namely “benign” job demands and “malignant” ones. Specifically, job demands should be classified as one of two demands, challenge or hindrance, both of which have different effects on employees’ cognitive wellbeing (Cavanaugh et al., 2000).

Existing research on the mechanism of job demands affecting employees’ cognitive wellbeing focuses mainly on the work domain itself (LePine et al., 2005). Such studies do not integrate work and family domains. Despite the division seen in research, the two important social subsystems, namely, work and family, are inseparable; interaction between work and family can directly impact employees’ cognitive wellbeing (Voydanoff, 2005; Li et al., 2015). Based on the conservation of resources (COR) theory (Hobfoll, 1989), the outcome of a stressful event depends on whether the event results in a net gain or loss of resources (Cavanaugh et al., 2000). In line with this point, the pressures felt by employees in the workplace will inevitably affect their family life, whether positively or negatively. Numerous studies have confirmed that job demands may act as antecedent variables affecting work–family enrichment (Voydanoff, 2005; Wayne et al., 2007). However, the mechanism of how different types of job demands affect cognitive wellbeing requires further examination. Thus, it is necessary to undertake research confirming the importance of work–family enrichment in the process mechanism of transformation from employees’ hindrance and challenge demands to their cognitive wellbeing.

Moreover, the characteristics of employees’ personal perceptions can affect their cognitive wellbeing, among which overqualification plays a crucial role (Verhaest and Omey, 2006; Liu and Wang, 2012; Zheng et al., 2015). In terms of the theory underpinning this assumption, LePine et al. (2005) pointed out an individuals differentiated characteristics would result in different reactions to job demands. Regardless of challenge or hindrance demands, there remains an issue as to whether an individual has the ability and qualification to cope with the demands of a job. In other words, employees with different levels of perceived qualifications will express different responses to challenge and hindrance demands. As a result, one could consider the question, “what kind of person is more likely to overcome challenges of challenge or hindrance demands?”. Those who perceive their own academic knowledge, skills and experience as exceeding the requirement for his/her particular position (Chen et al., 2017) are able to more easily cope with challenge or hindrance demands. Conversely, employees with low levels of perceived overqualification will struggle with job demands. It is commonly understood that different individuals possess varying levels of perceived qualification in the workplace. Some employees may think their abilities are far beyond the demands for the job, while others will believe that their abilities are merely suitable for the position or that their abilities do not meet the job requirements (Chu and Wang, 2019). Such appraisals of one’s ability and position held will not only affect an employee’s work performance (Liang et al., 2019), but also have implications for the employee’s family life as matters spillover from work to family life (Hu et al., 2015). Ultimately, an employee’s cognitive wellbeing could be affected (Johnson et al., 2002). Thus, it becomes crucial to consider the moderating effect from the perception of overqualification at the individual level as it relates to the relationships between daily job demands, work–family enrichment, and employees’ cognitive wellbeing.

Most research on wellbeing uses only cross–sectional data (Diao et al., 2019), thus potentially overlooking fluctuations in job demands and employees’ wellbeing on a daily basis. Zhang et al. (2017) suggested that researchers should consider daily variations when examining the effect of job demands on employees’ attitudes and behaviors, because factors such as unexpected stressors encountered during workdays (Ilies et al., 2007; Rodell and Judge, 2009), emotional events in the workplace (Dimotakis et al., 2011), and work strategies and cognition (Judge et al., 2009) can result in fluctuations in employees’ wellbeing. Therefore, building dynamic models while also considering individual differences and intraindividual fluctuations is necessary (Koopmann et al., 2016).

**FIGURE 1:**
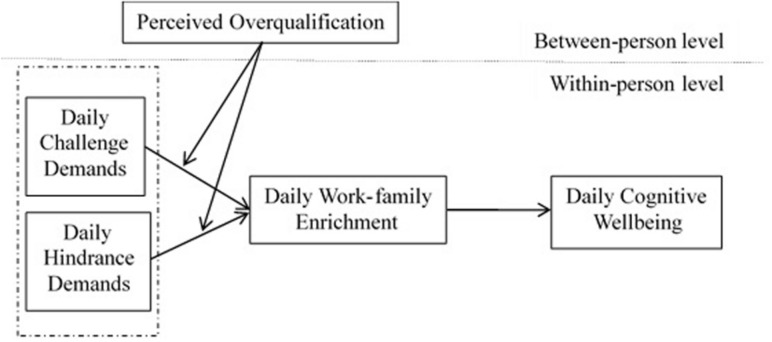
Theoretical research model.

This study is based on the challenge-hindrance stressor model and the COR theory and aims to analyze the ways in which challenge and hindrance demands affect employees’ wellbeing on a daily basis (this research model is shown in Figure 1). First, this study explores the predictive effect of employees’ daily challenge and hindrance demands on their occupational health, specifically on cognitive wellbeing. This aspect of the study is in response to existing research recommendations advocating the distinction between the two types of job demands (O’Brien and Beehr, 2019). Moreover, this study attempts to prove that challenge demands differ fundamentally from various hindrance demands (LePine et al., 2005). Second, daily work-family enrichment is introduced as a mediator. From the perspective of cross–border role participation, this study attempts to uncover the links between the two types of job demands and employees’ cognitive wellbeing. At the same time, this study seeks to enrich the literature on the two types of job demands as antecedent variables of work–family enrichment. Third, the employment of perceived overqualification as a moderating variable enables this study to explore various conditions from the individual differences to establish relationships between the two types of job demands, work–family enrichment, and employees’ cognitive wellbeing based on personality traits. Finally, this study utilizes the diary study method as a framework for considering the impact of variable changes, grasping the fluctuation trends of individuals, and reducing common method variance. Such an approach may better explain the effects of the variables and address the shortcomings of past empirical analyses.

## Theory and Hypotheses

### Challenge/Hindrance Demands and Cognitive Wellbeing

Challenge demands refer to employee job requirements that enable them to acquire new knowledge and result in the promotion of personal growth. Such requirements may include time pressure and increased work responsibilities. Challenge demands derive from specific daily work situations and are important external fluctuating predictors of employees’ cognitive wellbeing (Ilies et al., 2007; Rodell and Judge, 2009). According to the challenge hindrance stressor model, overcoming challenges means a potential net gain of resources. Thus daily challenge demands can prompt employees to devote increased energy to their work as a means of achieving their set work goals (LePine et al., 2005) and improving their satisfaction toward their daily cognitive abilities for the acquisition of greater resources. Second, these types of job demands can promote employees’ sense of control over their work, thereby enhancing the cognitive performance quality of their daily tasks. Finally, such demands can influence employee’s positive perception of their cognitive and intellectual abilities (Wu, 2000). This study maintains that daily challenge demands can significantly improve employees’ daily cognitive wellbeing.

Hypothesis 1: Daily challenge demands are positively related to daily cognitive wellbeing.

By contrast, hindrance demands refer to stressful and threateningly negative job requirements, including organizational politics, ambiguous roles, and conflict demands in the workplace. These qualities can potentially hinder personal career development and the achievement of work goals (Korunka et al., 2009; Anja et al., 2010). According to the challenge hindrance stressor model, employees consume vast amounts of emotional and cognitive resources when dealing with daily hindrance demands, thereby leading to work burnout (Prem et al., 2017). Moreover, role ambiguity and complex tasks generated by hindrance demands can result in a low sense of self–efficacy among employees in their daily work (e.g., information processing and/or decision making). Such loss in resources can weaken the individual’s sense of job competence (Huang and Peng, 2015). At the same time, employees tend to experience negative emotions when they lack control over their work (Song et al., 2011). Therefore, this study suggests that daily hindrance demands significantly reduce employees’ daily cognitive wellbeing.

Hypothesis 2: Daily hindrance demands are negatively related to daily cognitive wellbeing.

### Mediating Role of Work–Family Enrichment

Work–family enrichment emphasizes individuals’ contributions to the development of a social system (e.g., family or work) from their investment in that social system (e.g., work or family), including work–family enrichment and its opposite, family–work enrichment (Voydanoff, 2005; Tang et al., 2007; Wayne et al., 2007; Lin et al., 2013; Li et al., 2015). Existing research demonstrates that work–family enrichment is influenced by resource–based variables and job demands (Voydanoff, 2005; Wayne et al., 2007). Engaging in challenge demands can stimulate employees’ positive motivations, internal motivations, and sense of autonomy as well as accomplishment. Furthermore, it may help them better fulfill their family roles with positive emotions. At the same time, acquired skills can directly aid employees in solving family–related problems (Wayne et al., 2007; Anja et al., 2010; Yu and Zhang, 2018). Based on these findings, we believe that employees’ daily challenge demands can promote their daily work–family enrichment.

In contrast to what may be referred to as “benign” challenge demands, hindrance demands can negatively affect the balance between different cross–border roles and trigger negative emotions among employees. Thus, hindrance demands can ultimately lead to a rise in avoidance behaviors and dismission (Selye, 1956; Anja et al., 2010) and exert a negative impact on work–family enrichment.

Hypothesis 3: Daily challenge demands are positively related to daily work–family enrichment.

Hypothesis 4: Daily hindrance demands are negatively related to daily work–family enrichment.

Daily work–family enrichment can significantly improve employees’ daily cognitive wellbeing. As a subcategory of work wellbeing, cognitive wellbeing reflects the quality of employees’ cognitive effectiveness in the workplace and individuals’ perceptions of their perceptual behaviors (Dierendonck, 2004; Horn et al., 2004; Huang, 2014). According to Wayne et al. (2007), work–family enrichment can improve performance across work and family systems. When employees realize that their daily work will benefit not only their career advancement, but also their family happiness, they will experience increased positive emotions, self–efficacy, and work identification (van Steenbergen et al., 2007; Allis and O’Driscoll, 2008; Li et al., 2015). It is believed that these factors will positively affect an individual’s daily cognitive wellbeing.

Hypothesis 5: Daily work–family enrichment is positively related to daily cognitive wellbeing.

Hypothesis 6: Daily work–family enrichment mediates the relationship between daily challenge demands and daily cognitive wellbeing.

Hypothesis 7: Daily work–family enrichment mediates the relationship between daily hindrance demands and daily cognitive wellbeing.

### Moderating Role of Perceived Overqualification

Perceived overqualification refers to an individual’s perception that his/her academic knowledge, skills, and experiences are higher than those required for his/her position (Verhaest and Omey, 2006; Liu and Wang, 2012; Liu et al., 2015). Differences in employees’ perceived overqualification are expected to moderate the relationship between daily job demands (challenge demands and hindrance demands) and daily work–family enrichment.

Individuals with a high perceived overqualification generally show signs of high self–efficacy, high self–control, and high self–esteem. Such individuals tend to hold the belief that an increase in the tasks they take will correlate with additional work-related resources. Subsequently, they are willing to work hard to fulfill challenge demands (Luksyte et al., 2011; Maynard and Parfyonova, 2013). Such positive work experiences can enhance the positive effect of daily challenge demands on the individual’s daily work–family enrichment.

By contrast, employees with a low perceived overqualification can be characterized by the way in which their internal motivation to seek challenging work actively is insufficient; such employees can be noted for their belief that their personal abilities and skills simply match their position (Liu et al., 2015). When faced with challenge demands, this kind of employee is less confident about their abilities and may fear that failure to meet high challenging demands will result in resource loss. What is worse, with limited time and energy, they have less energy to devote to their family. Thus, we hypothesize that perceived overqualification strengthens the positive impact of daily challenge demands on daily work–family enrichment.

Hypothesis 8: Perceived overqualification moderates the relationship between daily challenge demands and daily work–family enrichment; thus, the relationship between the two elements will be strong in individuals with a high perception of overqualification.

The results differ with regards to daily hindrance demands. For employees possessing a high perceived overqualification, their perception of their abilities can make up for their boredom and aversion to emotional experiences that impede daily hindrance job demands (Erdogan and Bauer, 2009; Luksyte et al., 2011; Chu and Wang, 2019). Furthermore, their perception of the potential loss of work-related resources and its negative spillover effect on family domain will increase the balance between the energy and time they allocate to their work and family. This will also reduce the negative impact of the workplace on their family life (Verhaest and Omey, 2006; Liu and Wang, 2012). In addition, the negative impact between daily hindrance demands and daily work–family enrichment will also be reduced.

However, employees with a low perceived overqualification believe that their abilities match their jobs and that additional, complicated, and trivial hindrance demands entail extra energy and time consumption (Evans et al., 2009). With limited individual resources, they struggle to balance work and family. Moreover, it may result in an aversion to unstructured and routine work (Erdogan and Bauer, 2009; Luksyte et al., 2011), which in turn can increase the negative correlation between daily job demands and work–family enrichment.

Hypothesis 9: Perceived overqualification moderates the relationship between daily hindrance demands and work–family enrichment; thus, the relationship between the two elements will be strong in individuals with a low perception of overqualification.

### Moderated Mediating Effect

Based on the above inferences, this study discusses the moderating effect of perceived overqualification on the mediating effects of daily work–family enrichment. When faced with daily challenge demands, employees with a high perception of overqualification can strengthen the conversion of daily challenge demands to daily cognitive wellbeing through daily work–family enrichment. Daily high challenge demands will enable employees with an increased perception of overqualification to experience positive moods during their daily work because of the potential net gain of resources that accompanies processing challenging tasks. To such an individual, these types of tasks are believed to provide them with new opportunities to acquire new skills and knowledge and overcome boredom and aversion to perceived overqualification in their present work. In addition, positive emotions from coping with work challenges will likely increase work–family enrichment and cognitive wellbeing. Meanwhile, daily low challenge demands have overt connection to learning and development opportunities that can increase resources, thus they tend to handle the tasks in an ordinary manner calmly. In such contexts, low challenge demands exist as well as a low overflow effect from employees’ work to their family life. In the context of low perceived overqualification, employees cannot cope with challenges posed by high challenge demands, thereby reducing their daily work–family enrichment and further decreasing their daily cognitive wellbeing.

Similarly, for daily hindrance demands, individuals with a high perceived overqualification are less likely to experience their negative effect through daily work–family enrichment and will also have a reduced negative effect on their daily cognitive. Specifically, having a high level of perceived overqualification will give individuals sufficient energy to deal with complex transactional work and weaken negative emotional experiences. However, low hindrance demands mean less complicated and tedious routine tasks, low negative psychological experiences among employees, and a low possibility of development opportunities being blocked. Such situations are less prevalent in employees with a high perception of overqualification; thus, negative effects on work–family enrichment and further effects on cognitive wellbeing are reduced. For employees with low perceived overqualification, complex routine tasks and negative work experiences from high hindrance demands are magnified; the fear of resource loss in turn can intensify negative effects on work–family enrichment and cognitive wellbeing.

Hypothesis 10: Perceived overqualification moderately mediates the relationships between daily challenge demands, daily work–family enrichment, and daily cognitive wellbeing.

Hypothesis 11: Perceived overqualification moderately mediates the relationships between daily hindrance demands, daily work–family enrichment, and daily cognitive wellbeing.

The theoretical research model is presented in Figure 1.

## Materials and Methods

### Participants and Procedures

To capture daily work–family enrichment and changes in cognitive wellbeing, we employed the diary study method to test the theoretical model. People from China with a specific work background and working an average of 40 h a week were selected as the objects of this study. We released questionnaires through social media to encourage interested employees to participate in and share the questionnaire information. All the subjects volunteered to participate in this questionnaire survey, and we provided gifts worth approximately 100 yuan to them as compensation for completing the survey. In terms of sample selection, we strictly controlled for the working background of the subjects, all of whom are full-time employees who work an average of 40 h per week. Prior to data collection, the researchers of the project first trained participants and explained the purpose of the study and the data collection procedures. After the beginning of the survey, two researchers with this project issued questionnaires to the subjects at 16:00 every day. Participants were then reminded to complete the questions before 22:00 at 20:00 and were also asked to give feedback to the researchers after completing the questionnaire. These measures were taken in order to ensure that the subjects could complete the questionnaire on time. The participants’ fields of occupation included higher education (colleges and universities), finance, manufacturing, electronic networks, and other various industries. Most employees in these industries confront challenging work requirements and hindrance job requirements. Compared with other industries, most of these positions have certain entry requirements for education, knowledge, experience, and abilities, consistent with the definition of perceived overqualification. Data for this study were collected over a period of 12 working days. Of the 105 questionnaires collected, 99 were valid, with 1,074 valid data points. Among the 99 participants, 42.20% were male, with an average age of 33.11 years and an average job tenure of 7.29 years. Moreover, 76.00% of the participants were married, and 60.90% had a university degree.

### Measures

The scales used in this study are all 5-point Likert scales, which are assigned 1-5 points from “strongly disagree” to “strongly agree.” Because the scale used is in English, we strictly follow the “back-translated” procedure in order to ensure the accuracy of Chinese scale expression.

#### Daily Challenge/Hindrance Demands

Daily challenge/hindrance demands were measured by the Rodell and Judge (2009) eight-item scale. A sample item from the daily challenge demands is, “I need high–level skills to finish the job today,” and a sample item from the daily hindrance demands is, “I need to go through all kinds of red tape to complete the work today.” A five–point Likert scale was used for scoring. The coefficient alpha of daily challenge demands was 0.82–0.91. This was due to data being collected over 12 days of daily challenge demands. The mean value (M) of the coefficient alpha of the daily challenge demands was 0.86. The coefficient alpha of daily hindrance demands was 0.75–0.86, and the mean value (M) was 0.80.

#### Daily Work–Family Enrichment

Daily work–family enrichment was measured using the four–item measure of Wayne et al. (2004) on a five–point scale. A sample item from the scale is, “What I do at work helps me deal with personal and practical issues in my family life today.” The coefficient of the scale was 0.87–0.94, with a mean value of 0.89.

#### Daily Cognitive Wellbeing

Horn et al. (2004) posited that cognitive wellbeing is an important dimension of work wellbeing and thus developed a relative scale to measure cognitive wellbeing. Based on that initial research, Huang (2014) further developed a cognitive wellbeing scale for use with Chinese samples. Therefore, daily cognitive wellbeing was operationalized as an instantaneous dimension of cognitive wellbeing. The measure included five items scored on a five–point scale. A sample item from the scale is, “I can easily focus myself today.” The reliability of the scale was 0.91–0.92, with a mean value of 0.91.

#### Perceived Overqualification

Perceived overqualification was measured using a scale developed by Maynard et al. (2006). The scale measured perception of education, knowledge, experience, and excess ability and considered these as a unified whole, containing nine items scored on a full five–point scale. The Chinese translation used in this study was obtained from Yang (2014). The coefficient alpha of the scale was 0.76.

#### Control Variables

Given that work–family enrichment and cognitive wellbeing may be affected by age, gender, education level, and job tenure (Lapierre et al., 2017), these four factors were controlled in the model.

### Analysis

The data were multilevel and nested; thus, we used Mplus 6.11 (Muthén and Asparouhov, 2011) to conduct multilevel path analysis. The data were relatively complete, with a low missing rate. Therefore, SPSS was adopted to process the missing values.

## Results

### Multilevel Confirmatory Factor Analysis (MCFA)

We referred to Sonnentag et al. (2012) to test the MCFA method. First, we used MCFA to merge the variables gradually and examine changes in fitting degrees to test the discriminant validity of the model. The fitting indices of the five–factor model were satisfactory: χ^2^ = 346.21, *df* = 135, CFI = 0.96, and RMSEA = 0.04. The fitting indices of the model in the MCFA are shown in Table 1.

**TABLE 1:** Multilevel Confirmatory Factor Analysis.

Model	χ^2^	*df*	SRMR	RMSEA	CFI	TLI	AIC
Five factors model a	346.21	135	0.04	0.04	0.96	0.95	41145.92
Four factors model b	1039.15	143	0.09	0.08	0.83	0.80	42173.13
Three factors model c	2381.65	245	0.17	0.09	0.67	0.62	42247.87
Two factors model d	3941.52	246	0.17	0.12	0.42	0.35	44340.76
One factors model e	6937.71	437	0.31	0.12	0.00	–0.12	46158.58

*a = challenge demands; hindrance demands; perceived overqualification; work-family enrichment; cognitive wellbeing;b = challenge demands + hindrance demands; perceived overqualification; work-family enrichment; cognitive wellbeing; c = challenge demands + hindrance demands + perceived overqualification; work-family enrichment; cognitive wellbeing; d = challenge demands + hindrance demands + perceived overqualification; work-family enrichment + cognitive wellbeing; e = challenge demands + hindrance demands + perceived overqualification + work-family enrichment + cognitive wellbeing.*

### Descriptive Statistical Analysis

In this study, perceived overqualification was at the between–person level, whereas challenge demands/hindrance demands, work–family enrichment, and cognitive wellbeing were at the within–person level. The control variables (i.e., age, gender, education level, and job tenure) were included in the model as between–person–level variables. The descriptive statistics and correlation coefficient matrix of each variable are presented in Table 2.

**TABLE 2:** Descriptive Statistics and Correlations at both Between- and Within- Person Levels.

	M	SD	1	2	3	4	5	6	7	8	9
1. Gender	1.57	0.49	1	–0.04	0.01	−0.09**	−0.09**	0.03	−0.13**	−0.20**	0.07*
2. Age	33.11	7.15	–0.04	1	−0.15**	0.86**	–0.02	−0.07*	0.11**	0.20**	−0.12**
3. Educational level	4.33	1.10	0.01	−0.15**	1	−0.11**	−0.07*	–0.03	0.11**	0.02	0.08**
4. Job tenure	7.29	7.41	−0.09**	0.86**	−0.11**	1	0.01	–0.00	0.08*	0.16**	–0.00
5. Challenge demands	3.07	0.61	−0.12**	–0.03	−0.09**	0.01	1	0.46**	0.15**	0.16**	–0.02
6. Hindrance demands	3.36	0.91	0.05	−0.10**	–0.04	–0.00	0.54**	1	−0.11**	–0.04	0.11**
7. Work-family enrichment	3.65	0.69	−0.16**	0.14**	0.14**	0.10**	0.22**	−0.17**	1	0.35**	−0.07*
8. Cognitive wellbeing	3.31	0.84	−0.29**	0.29**	0.03	0.23**	0.13**	−0.20**	0.48**	1	−0.06*
9. Perceived overqualification	2.72	0.77	0.07*	−0.12**	0.08**	–0.00	–0.03	0.16**	−0.10**	−0.09**	1

Correlations below the diagonal are at the between-person level (N = 99); correlations above the diagonal are at the within-person level (N = 1074).*p < 0.05, **p < 0.01, ***p < 0.001.

Before testing the hypotheses, we examined the variations in daily work–family enrichment and daily cognitive wellbeing across levels. Table 3 shows that within–person variance in work–family enrichment was 0.30, accounting for 45% of the total variance, and between–person variance was 0.37, accounting for 55% of the total variance. Within–person variance in cognitive wellbeing was 0.27, accounting for 57% of the total variance, and between–person variance was 0.20, accounting for 43% of the total variance. Overall, although the amount of within-person variance was smaller than the amount of between-person variance for work-family enrichment, the above results These results indicated that significant variance at the between-person level for these variables (James, 1982). Thus, using a multilevel model was appropriate.

**TABLE 3:** Variance Components of the Null Model.

Variables	Within-person variances(e^2^)	Between-person variances(r^2^)	Percentage of between-person variances
Work-family enrichment	0.30**	0.37**	55%
Cognitive wellbeing	0.27**	0.20**	43%

e^2^ is the within-person variances in a variable; and r^2^ is the between-variances in the variable. The percentage of between-person variances was computed as r^2^/(e^2^+r^2^).*p < 0.05, **p < 0.01, ***p < 0.001, N = 1074.

### Hypothesis Testing

We employed the multilevel structural equation modeling approach to test the between– and within–person effects between the variables (Preacher et al., 2010). At the within–person level, challenge demands had a positive effect on employees’ cognitive wellbeing, whereas hindrance demands had a negative effect on employees’ cognitive wellbeing. The results seen in Table 4 show that daily challenge demands at the within–person level are able to positively predict employees’ daily cognitive wellbeing (γ = 0.19, *p* < 0.01), and daily hindrance demands at the within–person level negatively predict employees’ daily cognitive wellbeing (γ = −0.14, *p* < 0.05), both of these factors were consistent with Hypotheses 1 and 2.

**TABLE 4:** Multilevel Structural Equation Model of Direct Effect and Indirect Effect.

	Outcome					
	Daily work-family enrichment			Daily cognitive wellbeing		
	γ	SE	95%CI	γ	SE	95%CI
**Direct effect**						
Daily challenge demands	0.26**	0.07	[0.02,0.39]	0.19**	0.06	[0.07,0.31]
Daily hindrance demands	−0.25**	0.08	[−0.44, −0.10]	−0.14*	0.06	[−0.25, −0.02]
Daily work-family enrichment				0.29***	0.06	[0.18,0.41]
**Indirect effect**						
Daily challenge demands(through DWFE)				0.05**	0.02	[0.02, 0.08]
Daily hindrance demands(through DWFE)				−0.02	0.02	[−0.05,0.02]

*p < 0.05, **p < 0.01, ***p < 0.001, N = 1074.CI, confidence interval; DWFE, daily work-family enrichment.

Hypotheses 3 and 4 tested the effects between daily challenge and hindrance demands and work–family enrichment at the within–person level. The results in Table 4 indicate that daily challenge demands significantly predicted daily work–family enrichment (γ = 0.26, *p* < 0.01), with a 95% confidence interval of [0.02, 0.39], excluding 0. However, daily hindrance demands negatively predicted work–family enrichment at the within–person level (γ = −0.25, *p* < 0.01), with a 95% confidence interval of [–0.44, –0.10], excluding 0. Thus, these is support for Hypotheses 3 and 4.

At the within–person level, work–family enrichment was determined to have a positive effect on employees’ cognitive wellbeing. The results in Table 4 illustrate that daily work–family enrichment was positively correlated with employees’ daily cognitive wellbeing (γ = 0.29, *p* < 0.001), with a 95% confidence interval of [0.18, 0.41], excluding 0. Thus, Hypothesis 5 was supported.

Hypotheses 6 and 7 tested the mediating role of daily work–family enrichment. According to Table 4, daily challenge demands positively correlate with daily work–family enrichment (γ = 0.26, *p* < 0.01). Daily work–family enrichment positively correlates with daily cognitive wellbeing (γ = 0.29, *p* < 0.001), with a significant indirect effect (γ = 0.05, *p* < 0.05) and a 95% confidence interval of [0.02, 0.08], excluding 0. Thus, Hypothesis 6 is verifiable. Similarly, in Table 4, daily hindrance demands were negatively correlated with daily work–family enrichment (γ = −0.25, *p* < 0.001), with an insignificant indirect effect (γ = −0.02, n.s.) and a 95% confidence interval of [−0.05, 0.02] within 0. Thus, Hypothesis 7 was not verified.

**FIGURE 2:**
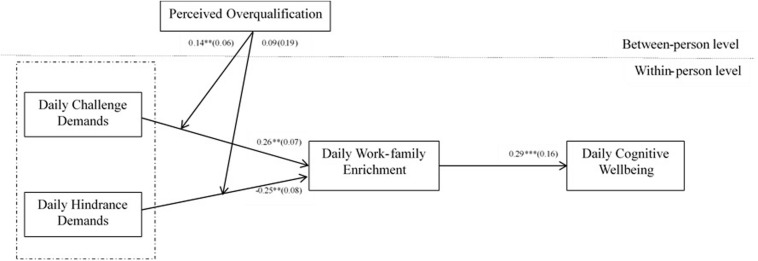
Results of multilevel path analysis. *p < 0.05, **p < 0.01, ***p < 0.001.

**FIGURE 3:**
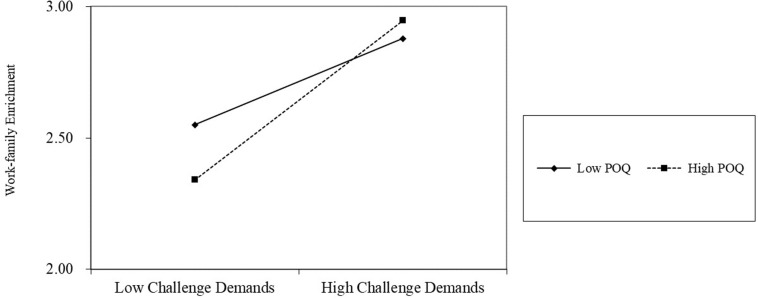
The moderating effect of perceived overqualification on the relationship between challenge demand and work-family enrichment at the within-person level.

Hypothesis 8 and 9 tested the moderating role of perceived overqualification (at the between–person level) on the relationship between daily challenge/hindrance demands on daily work-family enrichment (at the within-person level). The results seen in Figure 2 indicate that perceived overqualification positively moderated the relationship between daily challenge demands and daily work–family enrichment, with an interaction effect (γ = 0.28, *p* < 0.01). This result suggests that under a high level of perceived overqualification, the relationship between daily challenge demands and daily work–family enrichment was strong (γ = 0.32, *p* < 0.001), whereas under a low level of perceived overqualification, this relationship was not significant (γ = 0.05, n.s.). Thus, Hypothesis 8 was supported. The interactive effect of daily challenge demands and daily work–family enrichment is presented in Figure 3. Similarly, Figure 2 showed that perceived overqualification did not moderate the relationship between daily hindrance demands and daily work-family enrichment, with an insignificant interaction effect (γ = 0.09, n.s.) and a 95% confidence interval of [−0.04, 0.21] within 0. Thus, Hypothesis 9 was not verified.

Hypothesis 10 and 11 stated that perceived overqualification (at the between–person level) could moderate the relationships between daily challenge/hindrance demands (within–person level), daily work–family enrichment (within–person level), and daily cognitive wellbeing (within–person level). The results in Table 5 show the significant conditional indirect effects of perceived overqualification, specifically, at the between–person level on daily challenge demands and at the within–person level on work–family enrichment and cognitive wellbeing. The difference between high and low perceived overqualification was significant (*d* = 0.03, *p* < 0.05), with a 95% confidence interval of [0.00, 0.06], excluding 0. Thus, Hypothesis 10 was supported. However, no significant moderating effect was observed on hindrance demands, work–family enrichment, and cognitive wellbeing. Furthermore, a significant difference was observed between the two groups (*d* = 0.02, n.s.), with a 95% confidence interval of [−0.01, 0.05] within 0. Thus, Hypothesis 11 was not supported.

**TABLE 5:** Moderated Mediating Model.

Predictors	Mediator: Daily work-family enrichment			
	Moderators	Indirect effect	95% Lower	95% Upper
Daily challenge demands	High POQ	0.03	0.00	0.05
	Low POQ	0.00	–0.02	0.01
	Differences	0.03	0.00	0.06
Daily hindrance demands	High POQ	0.00	–0.03	0.02
	Low POQ	–0.02	–0.04	0.00
	Differences	0.02	–0.01	0.05

*POQ, perceived overqualification.*

## Discussion

Based on the challenge-hindrance stressor model, we used the diary research method to examine how the two types of job demands (i.e., challenge/hindrance demands) affect employees’ cognitive wellbeing while also giving consideration to the effect of work–family enrichment. Moreover, this study explores the moderating effect of overqualification on the relationships between employees’ daily challenge and hindrance demands, daily work–family enrichment, and daily cognitive wellbeing.

### Theoretical Contribution

The present study contributes to the existing literature as outlined below. First, this study further replenishes the research on challenge-hindrance stressor model by examining how two types of demand, namely challenge demands/hindrance demands, affect employee’s wellbeing. Existing research has concluded that work demand negatively influences employee’s work-family enrichment and well-being (e.g., Shimada et al., 2010; Proost et al., 2010). However, examining the effects of challenge demands and hindrance demands on employee’s work-family enrichment and cognitive well-being simultaneously is a more comprehensive approach. Although work demand places pressure on an employee, a challenge demand presents itself as a “good stressor” that allows for an employee to gain benefits from dealing with said challenge. The benefits may come in the form of personal growth or useful experiences in coping with work tasks. Regarding work demand as a whole to be negative runs the risk of engaging in overgeneralization. The research results presented here provide evidence that hindrance demand differs from challenge demand in nature. A hindrance demand is negatively related to work-family enrichment and cognitive well-being; however, challenge demand positively correlates with work-family enrichment and cognitive well-being.

Secondly, from the perspective of cross work-family interface, this study further refines the influencing mechanism of the hindrance/challenge demands on employees’ cognitive well-being. In addition, prior studies have demonstrated that work-family enrichment is a predictor of occupational wellbeing (Tang et al., 2014). However, occupational wellbeing is conceptualized in general, including psychological wellbeing and subjective wellbeing. Specially, subjective wellbeing consists of affective wellbeing and cognitive wellbeing (Luhmann et al., 2012a). Schimmack (2008) pointed out that affective wellbeing and cognitive wellbeing have different predictors in which domain (work domain or family domain) satisfaction has a stronger prediction on cognitive wellbeing than affective wellbeing. Compared to other type of wellbeing, cognitive wellbeing is more related to the perception of work-family interface. Therefore, studying the mediating role of work-family enrichment in bridging hindrance/challenging needs and cognitive well-being in the field of research on work-family relationship represents a more targeted approach. The conclusion of this study shows that daily work-family enrichment mediates the relationship between daily challenging demands and cognitive well-being. However, contrary to expectations, the mediating effects of work-family enrichment in the relationship between hindrance demand and cognitive well-being were not significant. The reason for this finding may stem from work–family enrichment involving employees’ positive perceptions of work and family relationships, thereby transmitting the effect of positive work demands on employees’ cognitive wellbeing. The meta–analysis results of Lapierre et al. (2017) indicated that “resource–providing contexts” have greater effects on work–family enrichment than “resource–depleting contexts.” The work presented here verifies the research conclusion of Lapierre et al. (2017) that work–family enrichment as a positive individual perception can easily mediate the relationship between resource–supply job demands (i.e., challenge demands) and cognitive wellbeing compared with that between resource–consuming job demands (i.e., hindrance demands) and cognitive wellbeing.

Thirdly, previous studies have explored the moderating effect of work resources on the relationship between challenge/hindrance demands on employee’s well-being (Tadić et al., 2015), but there remains room to consider its moderating effect between different work demands and cognitive well-being from the perspective of individual characteristics differences of employees. Starting with the question of who is more likely to overcome the challenges posed by challenging/hindrance work demands, we examined the moderating mediation effect of overqualification on the relationship among daily challenging/hindrance work demands, daily work-family enrichment and daily cognitive well-being. The results show that the relationship between daily challenge demands and daily work-family enrichment, and the indirect effect of daily challenging work demands on daily cognitive well-being through daily work-family enrichment were stronger in employees with a higher level of over-qualification. However, the moderating mediation effect of overqualification on the relationship among daily hindrance work demands, daily work-family enrichment and daily cognitive well-being were not significant. These results remind us that for employees with strong perceptions of overqualification, challenge demands could make up for the their inadaptation of overqualification. However, as a “negative” work demand, hindrance demands could decrease employee’s work-family enrichment and cognitive well-being, regardless of the employee being overqualified.

Fourth, this study uses the diary research method to collect data to reveal the dynamic mechanism of daily fluctuations in job demands on daily cognitive wellbeing. As a research method, cross–sectional data research is highly suitable for studying the effect of major events but inadequate for capturing the pressures of daily life (i.e., job needs; Zhang et al., 2017). Therefore, in response to the suggestions put forth by Helms and Demo (2005) to examine daily and dynamically fluctuating work pressures, this study noted that the two types of job demands will exhibit daily dynamic fluctuations in an individual and thus used the diary research method (Ohly and Fritz, 2010; Song et al., 2011). Since the requirements for challenging/hindrance work are determined by specific tasks and procedures in the work area each day, the work tasks will likely have a high demand for challenging/hindrance work on one day and a low demand for challenging/hindrance work on another (Butler et al., 2005).

### Practical Implications

First, enterprises should improve upon job design, increase challenging tasks and challenge motivations, reduce hindrance tasks, and provide work protection to prevent unnecessary resource consumption. At the same time, managers should also maintain an awareness that the range of demands for challenging work is a kind of pressure for employees, which negatively affects their attitudes and behaviors. Therefore, the question of how to maintain the demand for challenging work at a reasonable level is also a problem that managers need to consider.

Second, overqualification is a double–edged sword, as it can entail not only high performance but also high turnover rates, low job satisfaction and work commitment, and organizational citizenship behavior among employees. Thus, reasonable staff arrangements and matching posts for employees also play a crucial role in improving staff wellbeing. One of the purposes of human resource management is to fully mobilize the enthusiasm of employees and enhance their potential to create greater value for the enterprise, even beyond their respective job roles. Generally, a better match between the employee and the position allows for better outcomes for both the organization and employees themselves. Consistent with this point, matching posts for employees also plays a crucial role in improving staff wellbeing. Thus, managers should give thought to strengthening the rationality of staff arrangements. Also, regular feedback for an employee’s performance, and employee potential testing and development are necessary to increasing an employee’s position-person fit. Although, it is important to assign “challenging” but appropriate tasks to employees, seeking feedback on an employee’s perception of their own overqualification is also critical. For example, in terms of recruitment and probation period, managers should make a comprehensive consideration regarding personal characteristics and work ability to select the most suitable applicants as opposed to simply the most capable ones. Subsequently they may allocate employees to a more suitable position dynamically, according to his/her present experience and ability. In essence, the most appropriate employee is the best employee.

### Limitations and Future Direction

This study still faces the following limitations that require further improvement for the purposes of future research. First, all questionnaires in this study adopt self-evaluation, which is prone to common method deviation. Although this study has proved that the possible common method deviation is not serious according to the statistical results, such situations should still be avoided in future studies. In addition, daily variables are measured by a single point measurement. However, a multiple point measuring method would be more accurate, such as a measurement of challenge demand/hindrance during work time and measuring work-family enrichment and cognitive well-being during family time. We suggest that future research adopt a more rigorous research design to examine factors in the workplace for employee attitude and behavior in the field of family domain.

Second, this research mainly discusses the effects of two different types of job demand on employee’s work-family enrichment and cognitive well-being; however, this study suggests that challenging demand may have an effect on work-family enrichment through different channels. For example, challenging demand can improve staff work-family enrichment by an employee’s internal motivation, yet it could also have a negative impact by causing employee burnout in the course of coping with a challenge. We also encourage future studies to further test the mechanism of how challenge demand affects work family enrichment under the guidance of different theories. Besides, we only include cognitive wellbeing in our model, whereas there are other types of wellbeing (i.e., affective wellbeing, psychological wellbeing). Although cognitive wellbeing is more closely related to work-family interface satisfaction (Schimmack, 2008), work-family enrichment includes cognitive and affective effects. Future studies could focus on different dimensions of wellbeing and explore its predictors cross work and family domains.

Third, the samples of this study are all from China, but there remains a lack of consideration for Chinese-specific situation variables, such as leadership, membership and traditional Chinese culture. Thus, future studies may wish to further consider whether employees react differently in coping with challenge demand and hindrance demand in different cultural backgrounds. Such an investigation could yield promising results for management practitioners to manage cross-cultural differences. In addition, the targets’ job roles are also essential to their experience of perceived overqualification. Thus, future studies could consider the targets’ work background in a more comprehensive and nuanced way.

## Conclusion

The findings of current research shed light on how and when job demands influenced on employees’ cognitive wellbeing. Based on the challenge-hindrance stressor model, we demonstrated the opposite effects of challenge/hindrance demands on employees’ cognitive wellbeing through work-family enrichment. Furthermore, our findings suggested that individuals with perceived overqualification could cope with challenge demands better. Moreover, our results revealed it was necessary to employ the diary study to observe the daily fluctuation of job demands, work-family enrichment, and cognitive wellbeing, advancing current research by incorporating finer granularity. To sum up, balancing the work and family domains is an important issue for wellbeing. Meanwhile, we should focus on the individual difference in response.

## Data Availability Statement

The raw data supporting the conclusions of this article will be made available by the authors, without undue reservation.

## Ethics Statement

Ethical review and approval was not required for the study on human participants in accordance with the local legislation and institutional requirements. The patients/participants provided their written informed consent to participate in this study.

## Author Contributions

HC contributed to the conception of the study and wrote the manuscript. HW performed the data analyses and wrote the manuscript. MY contributed to analysis and manuscript preparation. SX performed the data collection and helped the analyses part. All authors contributed to the article and approved the submitted version.

## Conflict of Interest

The authors declare that the research was conducted in the absence of any commercial or financial relationships that could be construed as a potential conflict of interest.
